# Food hypersensitivity during SIT

**DOI:** 10.1186/2045-7022-5-S3-P72

**Published:** 2015-03-30

**Authors:** Jacek Gocki, Zbigniew Bartuzi

**Affiliations:** 1 Collegium Medicum, University of N. Copernicus, Bydgoszcz, Poland

## Background

In natural history of allergy, in some patients sensitized to aeroallergens, appear allergy to novel allergens, and in some cases this are food allergens. Subcutaneous immunotherapy (SIT) is the method of treatment patient with pollen allergy, and one of the aim of this treatment is protection against development novel allergy, but sometimes in clinical practice we observed hypersensitivity to new allergens, especially to food allergens.

## Aim

The aim of the study is assessment the prevalence of hypersensitivity to novel food allergens in patients treated SIT.

## Methods

We analysis case history 60 patients with SIT for aeroallergens, in age 18 to 72 years (medium 55 years), 31 women and 29 man.

## Result

Most patient were treated SIT due allergy to grass, and in the next place to dust mites, trees and weeds. In 5 (8,3%) patients of treated SIT we develop hypersensitivity to new food allergens. In 3 patients sensitized to trees we recognized hypersensitivity to apple, nectarine, peach, kiwi, carrot and hazelnut. In one patient sensitized to grass we develop hypersensitivity to pistachio nut, and in one patient sensitized to mugwort, hypersensitivity to shrimp was recognized. Oral allergy syndrome (OAS) was more common clinical manifestation - in 4 patients. One patient has rash on skin. All patient were treated SIT during 4 or 5 years.

## Conclusion

In our opinion in this cases of new hypersensitivity probably were provoked by SIT and this method therapy don't protect all patients with pollen allergy against development new allergy.

